# Delayed Recurrence of Acute Colonic Pseudo-Obstruction in the Setting of Acute Hypoxic Respiratory Failure

**DOI:** 10.7759/cureus.32079

**Published:** 2022-11-30

**Authors:** Richard J Gawel, Qian Zhang, Margot I Boigon

**Affiliations:** 1 Internal Medicine, Abington Hospital, Abington, USA

**Keywords:** gastrointestinal ileus, lumbar laminectomy, neostigmine, acpo, acute colonic pseudo-obstruction, ogilvie’s syndrome

## Abstract

Acute colonic pseudo-obstruction (ACPO) is a rare cause of massive colonic dilation without mechanical obstruction. We report on a 58-year-old gentleman who developed two separate episodes of ACPO following different surgical and medical stressors. The initial episode occurred shortly after lumbar laminectomy and was successfully managed with medical therapy. His second episode occurred several months later in the setting of acute hypoxic respiratory failure secondary to bacterial pneumonia and was refractory to conservative, medical, and endoscopic therapy. Recurrence and the refractory nature of symptoms are presumably multifactorial in etiology, likely due to his episode of acute hypoxic respiratory failure in the setting of chronic immobility following recent spine surgery. The patient was discharged in stable condition to a subacute rehabilitation facility with the expectation that physical therapy would improve his abdominal symptoms.

## Introduction

Acute colonic pseudo-obstruction (ACPO, also known as Ogilvie syndrome) is a rare cause of massive colonic dilation without mechanical obstruction and is most frequently seen in severely ill medical or surgical patients [1-4]. While most cases of ACPO resolve with conservative management, complicated cases can result in bowel ischemia, perforation, and peritonitis, with mortality rates as high as 44% [4-6]. Treatment initially consists of conservative management, including bowel rest and electrolyte stabilization, although refractory cases typically require colonoscopic decompression, neostigmine therapy, or surgical intervention [7]. Failure to respond to initial management is usually evident within the first 48-72 hours, prompting further intervention. Several case reports have documented the recurrence of ACPO in the weeks to months following the resolution of a previous episode, although each of these recurrent cases was in the setting of a similar stressor as that which precipitated the initial episode [8-11]. However, to our knowledge, no cases of recurrent ACPO have been reported following a completely different medical or surgical stressor. To increase awareness of the potential for delayed recurrence, we report a case of ACPO encountered during hospitalization for acute hypoxic respiratory failure several months after a previous medically responsive episode of ACPO during a prior hospitalization for spinal surgery.

## Case presentation

**Figure 1:**
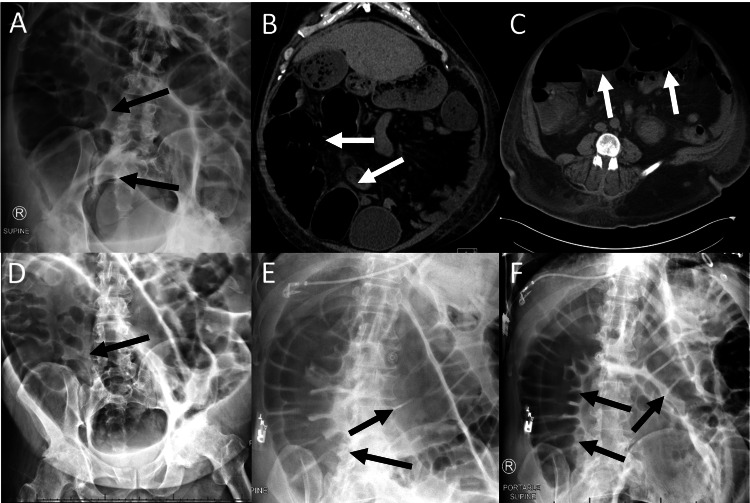
Imaging throughout the course of hospitalization. Initial plain film (A) showing cecal dilation to 12.6 cm. Coronal (B) and transverse (C) non-contrast computerized tomography images showing diffuse colonic and cecal dilation without evidence of mechanical obstruction. Plain film (D) three hours after the first neostigmine treatment showing a considerable reduction in colonic and cecal dilation to 6.2 cm. Plain film (E) 18 hours after the first neostigmine treatment showing recurrence of colonic and cecal dilation. Plain film (F) 20 hours after the second neostigmine treatment showing diffuse colonic and cecal dilation.

A 58-year-old man was admitted to the intensive care unit for acute hypoxic respiratory failure secondary to bacterial pneumonia, requiring non-invasive positive pressure ventilation. His medical history was significant for hypertension and gastroesophageal reflux disease, and he was persistently non-ambulatory following a recent lumbar laminectomy at another hospital. Respiratory status improved over the next week with antibiotics and supportive care, during which time the patient developed progressively worsening painless abdominal distension and incontinence of large volumes of non-bloody, watery diarrhea. Physical examination revealed considerable non-tender abdominal distension tympanic to percussion, with high-pitched bowel sounds. An abdominal x-ray demonstrated diffuse colonic and cecal dilation to 12.6 cm (Figure 1A), and non-contrast computerized tomography (CT) imaging of the abdomen confirmed colonic dilation without evidence of mechanical obstruction (Figures 1B, 1C). Evaluation for potentially offending infectious etiologies, including *C. difficile*, Shiga toxins, *Giardia* and *Cryptosporidium *antigens, *Histoplasma *antigens, and stool culture, was unrevealing. Neuroendocrine evaluation consisting of somatostatin, calprotectin, pancreatic elastase, tissue transglutaminase, and vasoactive intestinal peptide was also negative. The patient was noted to have elevated serum chromogranin A and serum gastrin, both of which were attributed to his hypertension and chronic use of proton pump inhibitors [12-14].

Review of the patient’s medical records from his recent hospitalization revealed that he developed similar abdominal distension 48 hours after an otherwise uncomplicated L2-L5 laminectomy. Based on CT imaging demonstrating significant colonic dilation without mechanical obstruction, he was diagnosed with ACPO. Not responding to conservative management, the patient received two treatments of intravenous neostigmine over the course of two consecutive days, followed by four days of oral pyridostigmine. His symptoms promptly resolved, and he did not experience any symptom recurrence during this time. He was eventually discharged to a skilled nursing facility for physical rehabilitation, where he had loose stools but no persistent abdominal distension. Of note, the patient was non-ambulatory throughout the course of his rehabilitation and upon admission to our hospital.

Given his recent ACPO diagnosis and lack of an alternative etiology to explain his present symptoms, he was diagnosed with recurrent ACPO and treated according to the standard of care [7]. Symptoms failed to improve after several days of conservative management consisting of diet restriction, electrolyte stabilization, and rectal tube placement. The patient underwent flexible sigmoidoscopy decompression, resulting in a near-complete resolution of symptoms, though abdominal distension gradually returned over the next 24 hours. He subsequently received two consecutive treatments of intravenous neostigmine over two days with only transient relief of symptoms (Figure 1D), followed by another endoscopic decompression with only temporary improvement. Abdominal distension and colonic dilation returned within 24 hours after each treatment (Figures 1E, 1F).

Throughout his hospitalization, the patient never displayed signs or symptoms of peritonitis or bowel ischemia. Due to the lack of response to medical therapy and the low risk for bowel rupture, our surgical and gastroenterology colleagues agreed that this persistent abdominal distension was likely primarily due to prolonged ambulatory dysfunction and that surgical intervention would not be beneficial. Given the low risk for perforation and the likelihood that symptoms would improve with increased mobility and ambulation, the patient was discharged to a subacute rehabilitation facility for physical therapy.

The patient’s gastrointestinal symptoms gradually improved over the next several weeks during physical therapy, and he did not require subsequent hospitalization related to ACPO. He had a cardiac event while at the rehabilitation facility two months after being discharged from the hospital and was unable to be resuscitated.

## Discussion

The incidence of ACPO following lumbar laminectomy is relatively high, occurring in 0.3% to 1.2% of patients [15,16]. The epidemiology of ACPO in patients with acute hypoxic respiratory failure has been less thoroughly investigated, though a recent cohort study reported an incidence rate of 1.6% among patients hospitalized for acute respiratory distress syndrome [17]. Our patient initially developed classic ACPO symptoms within 48 hours after lumbar laminectomy and was successfully treated with intravenous neostigmine. During a subsequent hospitalization for acute hypoxic respiratory failure, he developed another episode of ACPO refractory to both conservative management and multiple treatments with neostigmine and colonoscopic decompression. The reason for this delayed recurrence is likely a culmination of multiple factors, including his case of acute hypoxic respiratory failure due to bacterial pneumonia, a recent laminectomy complicated by prolonged immobility, and previous episodes of ACPO within the past two months.

The current pathophysiological understanding of ACPO involves dysregulation of peristalsis and colonic motility resulting from imbalances within the enteric autonomic nervous system, particularly aberrant parasympathetic suppression and sympathetic stimulation [18,19]. It is possible that inadvertent damage to lumbar autonomic fibers during laminectomy might have contributed to our patient's first ACPO episode. However, given the prompt resolution of this initial episode followed by treatment failure during the second episode, the degree to which autonomic fiber damage contributed to the symptom development or persistence of his second episode is unclear. Rather, we speculate that our patient’s recurrent ACPO was the result of a sympathetic surge associated with severe acute respiratory illness. The persistence of ACPO symptoms, despite multiple colonoscopic decompressions and neostigmine treatments, was most likely a result of his prolonged ambulatory dysfunction.

Few published reports have documented delayed recurrence of ACPO after previously successful conservative, medical, or endoscopic management. In one case, a 36-year-old woman who previously responded to endoscopic decompression for ACPO immediately after a cesarean section developed recurrence after a subsequent cesarean section, requiring hemicolectomy [8]. Another case involved a 33-year-old woman who developed uncomplicated ACPO after two consecutive cesarean sections, both cases of which were successfully managed conservatively [9]. Additionally, a 19-year-old developed a spontaneous recurrence of ACPO eight months after undergoing decompressive cecostomy for non-obstructive colonic dilation, likely in the setting of selective adrenergic dysautonomia associated with infectious toxoplasmosis [10]. In each of these previously reported cases, the subsequent ACPO episode was associated with a similar medical or surgical stressor as that which precipitated the initial episode. Furthermore, each of these patients was fully ambulatory throughout symptom recurrence and management. 

## Conclusions

We describe an unusual case of recurrent acute colonic pseudo-obstruction associated with separate surgical and medical stressors. Delayed recurrence and refractory nature of symptoms, in this case, were likely multifactorial in etiology, presumably resulting from an episode of acute hypoxic respiratory failure in the setting of prolonged immobility following recent lumbar spinal surgery. Although rare, clinicians should be aware of the possible recurrence of ACPO following a different surgery or critical illness. 
